# Organisation of testing services, structural barriers and facilitators of routine HIV self-testing during sexually transmitted infection consultations: a qualitative study of patients and providers in Abidjan, Côte d’Ivoire

**DOI:** 10.1186/s12879-023-08625-x

**Published:** 2024-02-27

**Authors:** Sokhna Boye, Alexis Kouadio, Amélé Florence Kouvahe, Anthony Vautier, Odette Ky-Zerbo, Nicolas Rouveau, Mathieu Maheu-Giroux, Romain Silhol, Arlette Simo Fotso, Joseph Larmarange, Dolorès Pourette, for ATLAS team, Georges Amani Elvis, Kéba Badiane, Céline Bayac, Anne Bekelynck, Marie-Claude Boily, Guillaume Breton, Marc d’Elbée, Alice Desclaux, Annabel Desgrées du Loû, Moussa Diop Papa, Clémence Doumenc-Aïdara, Eboi Ehui, Medley Graham, Kévin Jean, Abdelaye Keita, Arsène Kouassi Kra, Graham Medley, Raoul Moh, Tidiane Ndour Cheikh, Fern Terris-Prestholt, Mohamed Traore Métogara, Sanata Diallo, Alioune Gueye Papa, Olivier Geoffroy, Odé Kanku Kabemba, Armand Abokon, Camille Anoma, Annie Diokouri, Blaise Kouame, Venance Kouakou, Odette Koffi, Alain-Michel Kpolo, Josiane Tety, Yacouba Traore, Jules Bagendabanga, Djelika Berthé, Daouda Diakite, Mahamadou Diakité, Youssouf Diallo, Minta Daouda, Septime Hessou, Saidou Kanambaye, Abdul Karim Kanoute, Dembele Bintou Keita, Dramane Koné, Mariam Koné, Almoustapha Maiga, Telly Nouhoum, Abdoulaye Sanogo, Keita Aminata Saran, Fadiala Sidibé, Madani Tall, Camara Adam Yattassaye, Idrissa Bâ, Amadou Niang Diallo Papa, Fatou Fall, Fatou NGom Guèye NDèye, Sidy Mokhtar Ndiaye, Alassane Moussa Niang, Oumar Samba, Safiatou Thiam, Nguissali M. E. Turpin, Seydou Bouaré, Cheick Sidi Camara, Ehua Agnes Eponon, Marie-Anne Montaufray, Rosine Mosso, Pauline Dama Ndeye, Sophie Sarrassat, Souleymane Sow

**Affiliations:** 1 Centre Population Et Développement (Ceped), Institut de Recherche Pour Le Développement (IRD), Université Paris Cité, Inserm, France; 2 Université Felix Houphouët Boigny (UFHB), Institut d’Ethno-Sociologie (IES), Abidjan, Côte d’Ivoire; 3 Solidarité Thérapeutique Et Initiatives Pour La Santé (Solthis), Paris, France; 4 TransVIHMI (IRD, Université de Montpellier, INSERM), Montpellier, France; 5 Department of Epidemiology and Biostatistics, School of Population and Global Health, McGill University, Montréal, QC H3A 1A2 Canada; 6 MRC Centre for Global Infectious Disease Analysis, School of Public Health, Imperial College London, London, UK; 7 Institut National d’Études Démographique (INED), 9 Cr Des Humanités, 93300 Aubervilliers, France

**Keywords:** Self-testing, Screening, HIV, HIV testing offer, HIV self-testing, Sexually transmitted infections-STIs, Côte d’Ivoire

## Abstract

**Background:**

Consultations for sexually transmitted infection (STI) provide an opportunity to offer HIV testing to both patients and their partners. This study describes the organisation of HIV self-testing (HIVST) distribution during STI consultations in Abidjan (Côte d’Ivoire) and analyse the perceived barriers and facilitators associated with the use and redistribution of HIVST kits by STI patients.

**Materials and methods:**

A qualitative study was conducted between March and August 2021 to investigate three services providing HIVST: an antenatal care clinic (ANC), a general health centre that also provided STI consultations, and a dedicated STI clinic. Data were collected through observations of medical consultations with STI patients (*N* = 98) and interviews with both health professionals involved in HIVST distribution (*N* = 18) and STI patients who received HIVST kits for their partners (*N* = 20).

**Results:**

In the ANC clinic, HIV testing was routinely offered during the first prenatal visit. HIVST was commonly offered to women who had been diagnosed with an STI for their partner’s use (27/29 observations). In the general health centre, two parallel pathways coexisted: before the consultation, a risk assessment tool was used to offer HIV testing to eligible patients and, after the consultation, patients who had been diagnosed with an STI were referred to a care assistant for HIVST. Due to this HIV testing patient flow, few offers of HIV testing and HIVST were made in this setting (3/16). At the dedicated STI clinic, an HIVST video was played in the waiting room. According to the health professionals interviewed, this video helped reduce the time required to offer HIVST after the consultation. Task-shifting was implemented there: patients were referred to a nurse for HIV testing, and HIVST was commonly offered to STI patients for their partners’ use (28/53). When an HIVST was offered, it was generally accepted (54/58). Both health professionals and patients perceived HIVST positively despite experiencing a few difficulties with respect to offering HIVST to partners and structural barriers associated with the organisation of services.

**Conclusion:**

The organisation of patient flow and task-shifting influenced HIV testing and offers of HIVST kits. Proposing HIVST is more systematic when HIV testing is routinely offered to all patients. Successful integration requires improving the organisation of services, including task-shifting.

**Supplementary Information:**

The online version contains supplementary material available at 10.1186/s12879-023-08625-x.

## Introduction

Even though 85% of people living with HIV (PLHIV) worldwide knew their status in 2021, in West and Central Africa, diagnostic coverage was only 80% according to the *Joint United Nations Programme on HIV/AIDS (UNAIDS)* [1, 2]. HIV testing services remain an essential pillar if the goal of ending AIDS in this region is to be met by 2030. To increase HIV testing, particularly for people who have difficulties accessing health services, the World Health Organisation (WHO) recommends HIV self-testing (HIVST) as a complementary strategy [3]. HIVST is defined as a process by which the user collects a sample (oral fluid or blood), performs the test, and then interprets the result on his or her own, often in a private setting [3].

Since 2007, the WHO has recommended provider-initiated counselling and testing to routinise offer of HIV testing during medical consultations. This recommendation is particularly relevant for patients who have been diagnosed with a sexually transmitted infection (STI) and who can offer HIV testing to their partner(s) [4]. A study conducted in Malawi showed that testing the contacts of STI patients for HIV is an effective way to reach people who do not know they are living with HIV because HIV prevalence in this population was 3.2 times higher than among the contacts of other patients [5]. STI consultations provide an opportunity to promote HIV testing to both patients and their partners.

Like other West African countries, Côte d’Ivoire has adopted the WHO’s recommendations by encouraging routine HIV testing during STI consultations [6]. Despite these measures, certain challenges to routinising HIV testing during consultations for STI patients remain [7–9]. According to a general population survey conducted in 2017, less than one-third (28%) of people who had received an STI consultation in the last five years reported having been offered HIV testing [7]. In some clinics, the *President’s Emergency Plan for AIDS Relief* (PEPFAR) has implemented HIV rapid tests for patients with a certain risk score assessed by a short questionnaire. Before 2018, HIVST was not offered routinely in Côte d’Ivoire. Only some pilot studies have been conducted to investigate this topic, and they have not included HIVST in STI consultations.

Funded by Unitaid and coordinated by Solthis (Solidarité Thérapeutique et Initiatives pour la Santé), the ATLAS programme (*AutoTest-VIH, Libre d’Accéder à la connaissance de son Statut*) was launched in 2018 to promote and implement HIVST in Côte d’Ivoire, Mali, and Senegal. HIVST was integrated into existing testing policies in these countries, and 381,874 HIVST kits were distributed between July 2019 and December 2021 as part of the three countries’ national AIDS strategies.

In Côte d’Ivoire, ATLAS’ HIVST distribution was implemented through eight delivery channels (see Additional file 1). Five delivery channels adopted a facility-based strategy (delivery of HIVST in a health facility), and three used a community-based strategy focused on outreach activities. ATLAS' activities relied on both primary and secondary distribution. For secondary distribution, primary contacts (those who were in contact with distributors) were invited to redistribute HIVST kits to their peers, partners, and social network. Several research activities have been integrated into the program to document and support the programme’s implementation [10].

Documenting and understanding the introduction of HIVST during consultations by STI patients could help programmes adapt the organisation of HIV testing services in such settings. We conducted a comprehensive qualitative study of three services providing HIVST in Côte d’Ivoire (i.e., an antenatal care clinic, a general health centre, and an STI clinic). In this paper, we report on the organisation of HIVST distribution in the context of STI consultations and analyse the perceived barriers and facilitators associated with the distribution and use of HIVST kits.

## Materials and methods

### Study framework

As part of the introduction of HIVST during STI consultations, the Solthis implementation team working in Côte d’Ivoire conducted training sessions in 2019 to ensure the quality of HIVST distribution, in line with defined strategies and methods. This training was intended for health professionals who were either directly or indirectly involved in HIVST distribution with the goal of preparing them for the task of introducing HIVST during patient consultations. It focused on the role of health professionals and the practical aspects of HIVST distribution in the context of the ATLAS project (see all materials in Additional files 2, 3, 4, and 5, as well as documents available at https://atlas.solthis.org/categories_ressources/ressources-pratiques/). To facilitate the distribution of the HIVST kits to patients, all trained health professionals received the necessary materials. These materials included leaflets regarding the demonstration/use of the HIVST kits and the need to link to confirmatory testing (in case of reactive HIVST), as well as shareable demonstration videos (YouTube/WhatsApp) which were provided in French and translated into one of the local languages (Dioula). Information concerning health professionals’ participation in these training sessions is detailed in Table 3 (Additional file 6).

In line with WHO recommendations, the ATLAS protocol suggested that HIV testing should be offered to all patients who were diagnosed with an STI as well as their partner(s). If STI patients refused the HIV rapid test or if their partner(s) could not, or refused to, come to the health facility for rapid HIV testing, HIVST could be offered as an alternative strategy. Importantly, the patient flow and organisation of the HIV testing services were not prescribed in the ATLAS protocol: each service was free to organise its own services.

The manufacturer's instructions for use appeared insufficient when used on their own in a multilingual West African context [11]. To overcome such barriers, the ATLAS programme decided to develop locally-adapted brochures and explanatory videos in French and Dioula to help users perform the test, interpret the result and know what actions should be taken following a reactive or indeterminate result. They also encouraged people with a reactive HIVST to seek confirmatory HIV testing and care. Free phone lines have been set up in each country, and operators of these lines were trained about HIVST. Both manufacturer’s and programme’s tools were used on the field.

This qualitative study was conducted in Abidjan between March and August 2021 and focused on three different STI consultation services providing HIVST to patients and their partners.

 Service 1 was an antenatal care clinic (ANC) in an urban health centre that was located in a neighbourhood home and provided services to populations facing precarious economic situations. The clinic offered antenatal care, performed deliveries, and provided family planning services to women.

 Service 2 was a general health centre that served patients of all ages with various conditions. It was located in the same building as service 1. The centre offered different services, including HIV testing and STI consultations.

 Service 3 was a dedicated STI clinic. HIV testing was one of the main activities performed at this clinic.

### Data collection

Two qualitative approaches were used for data collection. First, observations of medical consultations with adult patients with symptoms of STIs were performed. Second, interviews were conducted with health professionals who were involved in distributing HIVST kits and patients who had received at least one HIVST kit for themselves and/or their partners.

 Data collection was performed by SB, a health anthropologist, and AK, a doctoral trainee in sociology. Both have several years of experience in the field of HIV and qualitative studies in West Africa. Data collection and analysis were supervised by DP, a senior health anthropologist.

 Observations of STI patients’ consultations were performed by the first and second authors (both authors performed the observations for the antenatal clinic (Service 1) and general health centre (Service 2), while only the second author performed the observations for the dedicated STI clinic (Service 3). They were allowed to access the consultation room when the health professional had reported an STI diagnosis and the patient's consent was obtained. These observations were recorded using a grid that collected patients’ personal information, such as their age and sex, as well as the time, duration, context, and setting in which the consultation took place. More detailed field notes were recorded using an observation guide (Additional file 7). These notes were related to interactions between healthcare workers and patients, the offer—or lack of offer—of HIV testing and/or HIVST for patients and/or their partners, and patients’ reactions. In addition, the anthropologists (investigators) had the opportunity to observe the patient flow more generally (in Services 1 to 3) and the interactions between the healthcare worker who was responsible for patient consultations and those who oversaw HIV testing in general (in Services 2 and 3).

A total of 98 patient consultations were observed: 29 at Service 1, 16 at Service 2, and 53 at Service 3. Details of the participants’ information were described in Table 1 in the Additional file 8.

The semistructured interviews consisted of one-on-one discussions between one of the investigators (the two first authors) and the participant. The participants included two categories: a) health professionals (doctor, nurse, midwife, care assistant, clinic counsellor, social worker) who were either directly or indirectly involved in the process of STI patients’ HIV testing and b) STI patients who were offered HIVST for themselves and/or their partner(s) during their consultation.

Our aim was to reach data saturation, i.e., to conduct interviews until no new information could be obtained. However, due to the unavailability of most of the people who benefited from the HIVST and their partner(s), we could conduct interviews only with individuals who were available and willing to participate in the study. Ultimately, a total of 38 individual interviews were conducted, including 18 interviews with health professionals and 20 with STI patients. The distribution and information of the participants are presented in Table 2 (Additional file 9) and Table 3 (Additional file 6).

The STI patients who participated in the study were contacted immediately after the consultations. After presenting the study to them, they were asked for their verbal consent to participate in the survey. If they agreed, they were offered an information leaflet, and we then proceeded to exchange telephone contact information to facilitate a subsequent call to schedule an interview appointment.

The interviews were conducted in French based on interview guides (Additional files 10, and 11). All interviews were recorded with the participant’s consent.

The first, second and last authors developed the interview guides based on the objectives and research questions of the study. The interview guides were tested at the beginning of the survey and readjusted according to the context.

The interviews with health professionals focused on their involvement in the management of STI patients, including HIV testing; the methods used to distribute HIVST kits to patients and their partner(s), the attitudes of STI patients towards the offer of HIVST kits, and health professionals’ perceptions and appreciation of HIVST (Additional file 10).

The interviews with the patients focused on the themes detailed in the interview grids (Additional file 11). Overall, these themes included patients’ knowledge of HIVST, their reactions to the HIVST distribution by the health professional, their partners’ reactions when they offered the use of HIVST, the use of HIVST (i.e., their understanding of the process as well as facilitating and complicating factors), and their perceptions of HIVST.

### Data analysis

All interviews were recorded in French and transcribed in full [12]. The data were pseudonymised before being coded and analysed using Dedoose qualitative data analysis software (www.dedoose.com). We conducted both thematic and inductive analyses. All the transcribed interviews were transferred into the Dedoose application, and then author 1, author 2 and the last author developed codes and subcodes based on the themes and questions developed in the interview guide. Other codes and subcodes were developed during the coding process by highlighting the themes that emerged from the interviews based on the data collected through content analysis [13, 14]. All observation notes taken during consultations with health professional were analysed using the same software [15]. A gendered approach was used to take the effects of gender into account in the data analysis [16].

### Ethical considerations

The study protocol, including the consent forms and procedures, was approved by the WHO Ethical Research Committee (07 August 2019, reference: ERC 0003181), the National Ethics Committee of Life Sciences and Health of Côte d’ Ivoire (28 May 2019, reference: ERC 0003181; 049–19/MSHP/CNESVS-kp), the Ethics Committee of the Faculty of Medicine and Pharmacy of the University of Bamako, Mali (14 August 2019, reference: 2019/88/CE/FMPOS), and the National Ethics Committee for Health Research of Senegal (26 July 2019, protocol SEN19/32). The study was conducted in accordance with the ethical conditions for this research. Consent was obtained from all participants in the survey. The investigators were required to respect the confidentiality of information, and the data were anonymised. To ensure the anonymity of the study sites, no identifying information was recorded when the services were presented.

## Results

### Study participants

The characteristics of the participants whose consultations were observed, and the participants with whom we conducted interviews are presented in Table 1, Table 2 and Table 3 in the Additional files 3, 4, and 5.

Of the 98 consultations that were observed across the three services, *N* = 29 were observed in the ANC clinic (Service 1), *N* = 16 were observed in the general health centre (Service 2) and *N* = 53 were observed in the STI clinic (Service 3).

In the ANC clinic, 1/3 of women were under 24 years of age.

In the general health centre, 2/3 of participants were women, and 1/3 were under 24 years of age.

In the STI clinic, more than ½ of participants were women and more than 2/3 were between 25 and 49 years of age. The majority of the participants in the three services were married or in a relationship (see Additional file 8).

The 18 health professionals interviewed were distributed as follows: 3 midwives in the ANC clinic (Service 1); 4 nurses, 3 doctors, 1 care assistant, 1 social worker, and 1 clinical counsellor in the general clinic (Service 2); and 3 doctors and 2 nurses in the STI-clinic (Service 3).

All healthcare staff working in Services 1 and 3 received either training on HIVST or on-site guidance from colleagues who had received such training. However, in Service 2, only 4/7 staff members who were responsible STI patients or involved in HIVST distribution had been trained in HIVST. These trained staff members included the clinic counsellor, a doctor, a social worker, and a nurse. Apart from these individuals, only the healthcare assistant responsible for HIV testing received on-site training, and the other staff members were ill-informed about the introduction of HIVST (see Additional file 6).

The 20 patient interviews were conducted at Service 1 (*N* = 5), Service 2 (*N* = 2), and Service 3 (*N* = 13). A total of 16/20 participants were women.

Patients were between 20 and 40 years old, and their level of education varied across the thee services. Across services, patients in the ANC clinic (Service 1) had lower levels of education (2 secondary and 3 primary level), while patients' education levels in the other two services were higher : 2/2 secondary in general health centre (Service 2); 2 secondary and 11 higher level in STI clinic (Service 3).

### Patient flow and the organisation of HIV testing

In this section, we describe the patient flow and organisation of the HIV testing offer, based on the observations performed in the three services and interviews with health professionals.

#### Antenatal care clinic (ANC)

In Service 1 (ANC), pregnant women were welcomed into the waiting area by a healthcare assistant who was responsible for taking their vitals (See Figure of patient flow in the Additional file 12). Four midwives conducted prenatal visits in two consultation rooms. In accordance with the national programme to prevent mother-to-child transmission, HIV testing was offered to almost all pregnant women during their first ANC visit. A midwife made the offer, and a rapid HIV test was performed directly during the consultation.

*No, here, we test (HIV testing) all women (…). At the first antenatal care visit, all women are tested. At the beginning, we had too many problems, there was reluctance, but now… they accept; if they don’t accept and they go to another centre, it’s the same thing.*

(Extract from interview with Midwife 2, Service 1)

Almost all the women who were diagnosed with an STI and whose ANC consultations were attended (*N* = 27/29) were offered an HIVST kit for their partner’s use. HIVST was generally offered directly without proposing that the partner could come to the health facility since, based on their experience, the midwives believed that the partner would otherwise refuse.

*Interviewer: With the self-test, when you propose it for the spouses, is it systematic, or do you first propose the conventional test? I mean, do you go through the woman by asking her if her husband can come, or do you propose the self-test directly?*

*Midwife: No, (…), we will give him an invitation, he will not come, so when we have (a patient with) an STI, we try to explain to the woman that your husband does not have time, maybe he comes home at night. Now, to do the HIV test, you don’t have to go anywhere, you don’t have to take a needle, you don’t have to have a health worker in front of you to do the test; he can do the test himself, and he doesn’t have to give me the result...*

(Excerpt from interview with Midwife 1, Service 1)

To demonstrate the use of the HIVST kits to patients, health professionals used a sample kit and instructions for use to describe the different steps with the help of supporting pictures. The average amount of time spent offering HIVST was 10 min. The midwives sometimes faced language barriers when the provider did not understand the patient’s language. As one of the midwives said,

*(...) although we explained, I explained in French, I explained in my twisted Dioula. The lady said yes, I understood. I took a sample in front of her. She said I understood the midwife. She left for home; she did not understand, she came back with someone....*

(Extract from interview with Midwife 3, Service 1)

#### General health centre

In Service 2 (the general health centre), medical consultations were conducted by three doctors and four nurses, who took turns: only two or three of these professionals worked on any given day. Care assistants were also present, who were responsible for welcoming patients and taking vitals in the waiting area before the consultations. One of the care assistants oversaw HIV testing activities. In the social service attached to the clinic, a clinic counsellor (a community worker who works in the clinic) and a social worker oversaw HIV testing and psychosocial follow-up for patients and PLHIV in particular. In terms of HIV testing, two patient flows were observed.

The first flow (flow A in Figure patient flow, in the Additionnal file 12) was already in place before the integration of ATLAS activities in August 2020. In the waiting area, before the medical consultations, the care assistant responsible for HIV testing was supposed to identify patients who were eligible for HIV testing using a risk/symptom assessment tool. Based on age, sex, sexual orientation, sexual practices, history of HIV testing, and potential symptoms, a questionnaire was administered to the patients to determine their eligibility for HIV testing. A rapid HIV test was administered in an office next door if the patient was eligible.

The second flow (flow B in Figure patient flow, in the Additionnal file 12) was introduced during the integration of HIVST activities in August 2021. During medical consultation, if an STI was diagnosed, the doctor or the nurse was supposed to offer HIV testing and propose a rapid HIV test or an HIVST to the patient. If the patient agreed, the patient was then referred to the care assistant responsible for HIV testing with a prescription or, if the care assistant was not present, to the social service. The doctor (or nurse) was also supposed to propose HIVST for the patient’s partner.

Among the 16 consultations with an STI patient observed in this context, HIV testing, including HIVST (2), was offered in only 6 instances and all 6 patients accepted the offer. Health professionals, especially nurses, did not systematically offer HIV testing to these patients.

Only 3/16 patients were offered an HIVST for their partner’s use. We observed a lack of coordination among the staff who were responsible for STI consultations and HIV testing. In addition, patients were not always referred to the care assistant or the social service for HIV rapid testing and/or for offers of HIVST kits for their partners’ use. One doctor and three nurses believed that it was the care assistant’s role to offer HIV testing to patients, while the care assistant regretted that patients were sometimes referred to her without her being notified that they had an STI (a condition that leads to an offer of an HIVST kit for their partners’ use).

*At her level, she (the care assistant) also has a questionnaire that she is supposed to administer to patients, which means that she does not execute HIV rapid tests for everyone. There is a questionnaire that is there, there are different entry points (…) Sometimes, even before the patient arrives here, she suggests the test, and when he agrees, she comes to ask us to make a report card, and then we make a report card for the person. She can screen without even knowing it is an STI.*

(Extract from interview with Doctor 3, Service 2)

The following excerpt from an interview with the care assistant confirmed the statement made by the doctor:

*Interviewer (I): Okay. Now in the case of STIs... and you, since you do the HIV testing (HIV rapid test execution), we also know that generally, sometimes patients don’t say so, but after consultations, the doctor may find that he has an STI or he himself talks about the points that make the doctor suspect an STI. In that case, does the doctor ever refer a patient to you because he has an STI?*

*Care Assistant (CA): Yes.*

*I: But when he refers the patient to you, does he just say, “He has an STI” or just “He must...”?*

*CA: No, he asks me to get the patient tested.*

(Excerpt from interview with a care assistant, Service 2)

Most health professionals (6/10) who were in contact with STI patients, including the care assistant who was responsible for HIV testing, had little or no awareness of, training in, or involvement in the offer of HIVST (see Additional file 6).

*Interviewer: You have not been trained, and have not had any coaching or any kind of make-up training?*

*Doctor: No, not at all. The first time I heard about self-tests was when the counsellor and a midwife went to Bassam in 2019 or 2020, I don’t even know. They had gone to Bassam as part of their training on self-testing, and then, thanks to you again, I heard about self-testing because when you came to speak, I drew the attention of the chief physician to ask, “Ah, but why are we being told about self-testing when they have not trained the people?” That’s when he told me that the people were trained in my absence because I was absent for four months (...) from the beginning of September until January (…). So, it was during that time that we had the self-testing training. When I came, well, I don’t know if there was any make-up training; well, I didn’t see any training report.*

(Extract from interview with Doctor 3, Service 2)

#### Dedicated STI consultations

In Service 3 (dedicated STI consultations), three doctors oversaw consultations for all patients. These doctors were assisted by two nurses who were responsible for taking the patients’ vitals, referrals, and STI screening. These doctors and nurses collaborated with a social service, which was also responsible for HIV testing and psychosocial follow-up with patients, especially PLHIV.

The patient flow (See Figure patient flow, in the Additionnal file 12) was reorganised to accommodate the integration of HIVST activities. Before the introduction of HIVST, the patient flow described was the same. However, the talks initiated by the clinic staff in the waiting room were performed without the accompanying HIVST video. In addition, the nurses were involved in the task of offering HIV testing, including HIVST.

First, videos on STIs, HIV testing in general and HIVST, in particular, were displayed in the waiting area. A presentation was held in this space every morning before consultations began. The talk was led by the clinic’s staff, including social workers, biologists, and nurses, under the supervision of the doctors who were responsible for patient consultations. The topics discussed during this activity were related to STIs,i.e., the definition, modes of transmission and measures for the prevention of STIs, as well as HIV testing, including HIVST.

*The activities generally start at 8 o’clock, and there are themes to which we pay particular attention, and these themes are related to STIs in general, all sexually transmitted diseases, whether it is the signs, prevention or testing, so we really try. Practically, we make a programme; each service has a time to come, and (we) give just a small conference to show either first the functioning of the structure and then why we have to communicate on STIs (...). The main objective is to have a transfer of some information outside the structure, which is a participative transfer and not a transfer that is done only within the structure (…). So, we review some themes a little bit, we ask the participants a little bit to bring their contributions, whether it is questions or contributions that really concern their experiences...*

(Extract from interview with Doctor 2, Service 3)

After the presentation, patients came to the nurses’ office. The nurse on duty determined whether the patient was in a condition to undergo some tests, particularly those related to biological STI screening, before referring them to the doctor.

*The sorting, we come here, at home here, and we ask him questions, if he hasn’t had sex for the last three days, or if it’s a woman, if she’s not indisposed (menstruating), if she doesn’t wash there thoroughly, if they’re not on antibiotics, things like that; that’s how we sort it out because if they’re not in the (appropriate) condition, we can’t take them to the doctor for the consultation.*

(Extract from interview with Nurse 2, Service 3)

The doctor saw the patient. All patients who were receiving an STI consultation for the first time were offered an HIV rapid test if they did not have proof of a recent test result. HIVST was also offered for use by the patient’s partner(s).

*We are an STI management service; it is inconceivable that a patient would come here with a proven STI syndrome and not be asked to take an HIV test (HIV testing offer for the STI patient).*

(Extract from an interview with Doctor 1, Service 3)

If the patient accepted, the doctor prescribed HIV testing for him and indicated the number of HIVST kits to be delivered according to the number of partners declared; the patient was then referred to the nurse. The nurse executed the HIV rapid test and/or delivered the HIVST kit(s) for the patient and their partner(s) according to the doctor’s prescription.

When HIV testing was offered to the patient, the rapid test offer was prioritised according to the relevant recommendations. HIVST was offered only when patients refused the HIV rapid test.

*HIV testing offer is mandatory because their entry point is an STI syndrome. An on-site HIV rapid test is what is prioritised; now, when the patient presents us with difficulties, such as “I am not psychologically ready, I am in a hurry”, instead of on-site testing (the HIV rapid test), we offer him the oral test (HIVST). And of course, if he has partners, we also offer him oral tests for his partners who could not be reached, who could not come here for STI visits, who do not send their partners here.*

(Excerpt from interview with Doctor 1, Service 3)

More than half of the patients (*N* = 28/53) were offered HIVST kits for their partners’ use. In addition, an invitation to bring their partner(s) for STI treatment, including HIV testing, was extended when the partner was not offered HIVST.

*Okay, that’s negative. Now if your honey is here, you have to go show her your result, it’s your passport. You have to tell her to come here and do it for her. You can’t force someone to take an HIV test, so you can’t force her to go there...*

(Excerpt from an exchange between a patient (woman) and Nurse 1 during the execution of an HIV rapid test)

Compared to Services 1 and 2, the health professionals working in Service 3 seemed to have less difficulty explaining the use of HIVST because the patients had already been introduced to this tool through the demonstration videos shown in the waiting room. When the patient was able to describe the use of HIVST from watching the HIVST demonstration video, the nurse was not required to demonstrate the process using the sample kit, which saved time.

*Interviewer: How do you explain it (the testing process) to him, because earlier you said that the man is a bit illiterate; how do you explain it to him?*

*Nurse: I ask him if he has been following the TV a little bit; if he says he has been following the TV, I say can’t you explain it to me a little bit. If he manages to explain to me: I say here, here; if he can’t, now I show him the instructions, I say here is this, how you have to do it, how you have to do your self-test and all that; if you eat, you have to wait 30 minutes, if you haven’t eaten also... generally it’s better to do it in the morning, if you haven’t brushed yourself; in any case, you do your self-test.*

(Excerpt from interview with Nurse 1, Service 3)

### Secondary distribution of HIVST kits to partners

#### Acceptability of the HIVST offer during medical consultations

According to the results, offering HIVST kits for partners’ use was received well by patients. In all three services, patients usually accepted the HIVST kits for their partners’ use when they were offered by health professionals. Of the 58 kits offered, only 4 were declined, either because the patients preferred to talk to their partner first, because they preferred to bring their partner to the health facility, or because they had already received an HIVST kit.

*I agreed to take a test; it is true that for me it is negative, but maybe for him, it may not be. That is why I agreed to take it so that he knows his status too.*

(Excerpt from interview with Patient 12, Service 3)

Almost all health professionals who were interviewed said that HIVST is generally accepted when it is offered to patients for their partners’ use.

*These patients react very well; they are surprised, aren’t they? They react very well; very few resist the tests. We have had some cases, but many accept it and take it, you see, they accept and take it, and they are curious to know how it works. It’s a new thing for them; they show interest in it.*

(Excerpt from interview with Doctor 1, Service 3)

We were able to conduct interviews with only 20/58 patients whose consultations were observed and who received an HIVST kit for them or their partner’s use (*N* = 5/27 in Service 1; *N* = 2/3 in Service 2; *N* = 13/28 in Service 3). Most patients had not yet offered HIVST kits to their partner(s), were unavailable or unreachable.

Almost all patients who received an HIVST kit for their partners’ use, and who agreed to talk to us, had offered the kit to their partner(s) by the time of the interview (*N* = 17/19); 13 partners accepted HIVST (although 3 had not used it at the time of interview and 3 did not know that it was an HIV test). Six partners had refused the HIVST kit, all of whom were men (Additional file 9, Table 2).

#### Patient’ s strategies for offering HIVST to partners

The majority (17/19) of patients interviewed who had received HIVST kits for their partners’ use were able to offer it to their partners, as demonstrated by this excerpt from a patient interview.

*Interviewer: But when your partners took the self-test and saw their results, what were their reactions?*

*A man: As I said, since I told them that I had already done my test, she also...there was one who had done her test before. So, she knew the result in advance, so it was a kind of confirmation. But for the other one, there was still enough joy.*

*Interviewer: Was there any resistance?*

*A man: No, no, not at all.*

(Excerpt from interview with Patient 3 service 3, who received 3 tests for partners)

However, proposing an HIVST to a partner is not always easy, especially for women. A few interviewees mentioned the fear associated with asking one’s partner to undergo testing for HIV. One woman told us about a trick she had used to get her partner to accept the test:

*When I arrived, I said “I had three things to give you” since I had the prescription which was very expensive. I didn’t give that first because that’s what I had to give last. So, when I arrived, I gave the test first. I said I have three things for you. This is you, your gift. I gave the test first and he took it (..) because he trusts me. He took to look at “what did I send”, and he took it. He read, he saw, I didn’t even speak, and he saw that... it was the HIV test. He asked me the question “where did I get that?” I said, “I had an appointment at the hospital here. I told you about it in the morning. So, when I came in, I did my test and they offered me to come and give you your test too.*

*He took it and then thanked me.*

(Excerpt from interview with Patient 6, Service 3)

Due to their hope of learning the HIV status of their spouse, a few women preferred not to disclose the purpose of the test to their partner, as illustrated by the following interview.

*Interviewer (I): And why, when he asked you “What kind of test is it?” did you not say “maybe it’s for HIV?” or “maybe it’s not?”*

*The woman: No, I thought if it’s HIV, if he comes here, maybe you’ll tell him.*

*I: Okay, you preferred not to say anything?*

*The woman: Maybe if I told him that’s it’s an HIV test, he wasn’t going to do it. (…)*

*He wasn’t going to do it because one day I came in for the test; I was pregnant with my fourth child. I came, they did the HIV test. They say it’s okay; they say there’s no problem, then to tell my husband to come. He didn’t come... He refused to come.*

(Interview extract with Patient 3, woman, Service 1)

Moreover, during our observations, one woman openly stated that she did not intend to reveal the test’s purpose to her husband, knowing in advance that he would not accept it if he knew.

*Midwife 1: But why are you laughing?*

*Woman: Laughing... I’m not going to tell him it’s an AIDS test...*

*Midwife 1, 2…: (Laughter in unison)*

*Midwife 1: But why?*

*Woman: If I tell him, he’s not going to want to do it... I’ll wait until after the results.*

*Midwife 2: But you don’t need to know the results (Laughs).*

*Midwife 1: But you’re going to give him my number; if he calls me, I’ll tell him. I’ll give you my number; I’ll write it down on a piece of paper.*

*Woman: (Laughs) I’ll explain it to him, and then I’ll tell him it’s an HIV test.*

(Excerpt from observation notes made during the provision of an HIVST kit to an STI patient during an ANC visit).

The wife ultimately seemed very relieved to be able to offer her husband an HIV test, noting that he had never agreed to be tested before. “I’m finally going to know my husband’s HIV status” she said when the HIVST kit was offered to her.

Other reasons, such as tensions within the couple and the fact that the partners did not live under the same roof, did not make it easier for patients to offer HIVST to their partner(s).

*Interviewer (I): Okay but when you gave him the oral test, did he do it?*

*Woman (W): (Respondent laughs) That’s the problem; I haven’t even given it to him yet.*

*I: Okay, but why?*

*W: That’s what I meant at the beginning; we’re not on good terms now. We haven’t seen each other for at least months, so...*

*I: But after the treatment, as you said earlier, did you talk to him about your treatment?*

*W: Yes, on the phone.*

*I: Oh, on the phone, but couldn’t you also suggest the oral test on the phone?*

*W: Uh...someone, you tell him something, he tells you that he is not infected, that he doesn’t have this kind of thing. How do you go about seeing him, getting him and offering him something else if he tells you he’s healthy?*

*I: So that’s what demotivated you to do it?*

*W: Yes, I tell myself that in three months, I can do it myself (respondent laughs), yes.*

(Excerpt from interview with Patient 7, woman, Service 3)

Other patients accepted the offer and managed to propose the use of HIVST to their spouse despite knowing, in advance that they would not accept it.

*Interviewer (I): Now, when you were given it (the HIVST kit), how did you react? Were you afraid to give it to your husband, or did you hesitate?*

*Woman: No, I’m not afraid to give it to him, but I knew he wouldn’t take it.*

(Excerpt from interview with Patient 5, Service 1)

Patients’ offers of HIVST to their partners could result in refusals: all observed cases of such refusal involved men (6/6). In contrast to men, women had more difficulty convincing their partners to accept HIVST, as the following extract shows.

*Generally, when a man agrees to take a traditional test (HIV rapid test) and when we suggest an oral test for his partner, he does not hesitate to take it, but it is in the other direction that the difficulty arises: “It is complicated, well, I don’t know how I’m going to say, oral test, he doesn’t have HIV, what is he going to do with that” (laughter) (...). There is one that we received this morning, she said, I gave the test to my spouse, but he refused to do the oral test, and she said as she had already done her test here, she told him that her test was negative. So, he said that means the result is the same, and the lady said, “No, the result may not be the same; you have to do your test”, and what did the lady say to us today? She said but since it’s a test that you put in the gums like this, I’m going to give him a sleeping pill, and when he’s asleep, I’m going to do his test (laughs). In fact, these are the realities that we live with, so it’s a bit like that, you see, that men don’t like to do their test, eh.*

(Excerpt from interview with Doctor 2, Service 3)

### Perceptions of HIVST by healthcare professionals and patients

Health professionals’ and patients’ perceptions of HIVST are diverse.

#### The positive perceptions of health professionals

Almost all healthcare professionals interviewed in the three services had positive perceptions of HIVST and believed that HIVST allows them to reach the partners of STI patients who were previously difficult to reach.

*It was difficult for us as health workers, especially the spouses, to get hold of them, but with the self-test, when we have cases of STIs, and the explanation is easy because in the kit there is a paper that shows us how to use it (...) The self-test also allowed me to get closer to some spouses, so some of them kept my number. Apart from the self-test, they often call me, “Ah, don’t you remember me? It’s my wife, I called you the other time for something (...) now my wife has this here, eh, madam, are you working this day, we want to come”. It has allowed me to get to know some spouses (...) there is a trust that has been built.*

(Extract from interview with Midwife 1, Service 1)

Several health workers, particularly in Services 1 and 3, noted that HIVST both facilitated the offer of HIV testing (both to the patient and for their partner’s use) and made it more systematic. Health professionals noted that it was more difficult to propose HIV testing before the introduction of HIVST. They had difficulty convincing patients’ partner(s) to come to the clinic. HIVST, therefore, was helpful to them, as illustrated by the following extracts from interviews with a midwife and a doctor.

*Yes, when we were in cases of determination, when it was repeated, I said to myself, “Ah, the spouse must be able to see clearly”, but with the self-test, when I see an STI, it is systematically offered.*

(Extract from interview with Midwife 2, Service 1)

*I think that self-testing is a godsend; I think that it facilitates care, and it allows the patient to be autonomous. That’s what we’re looking for nowadays in care, in all the specialties that we do nowadays, we ask that the patient be autonomous. So, if the patient himself can do his test and then interpret his result, I think that’s already good.*

(Extract from an interview with Doctor 3, Service 3)

Advantages related to ease of use, discretion, and safety were highlighted. In general, health professionals appreciated HIVST because it was easy, discreet, and limited the risk of accidents due to the absence of blood sampling.

*I think that yes, it’s good, because we don’t play with people’s blood (...) There is no injection, and it’s the person who puts the brush (spatula) on his gums. (...) There you are protected, so it’s welcome (...) I find the usage reliable (...) You don’t have to read; you are told to do it like this and like this, whereas with a needle stick, you are going to prick, and if the person moves, it can prick you, and you personally are exposed, whereas in this, you are not exposed (...) it also protects even the patient, because it can prick you and prick the patient, and then your bloods will cross while perhaps you are infected, so you infect him.*

(Extract from interview with Midwife 2, Service 1)

#### Some negative perceptions of health professionals

The ATLAS policy does not recommend the proactive and systematic tracking of HIVST's use and results: users could, if they wanted, report their test results to a dispensing agent, but such reporting should not be mandatory. Most health professionals misunderstood the policy. They understood they were not allowed to ask the patients about their HIVST use and results, and perceived that limitation negatively.

*I think that the self-test itself is better (laughs), only that it is the return, the determination, at least you yourself are there, you have seen, you can say, you yourself have done it, you have seen, but the self-test, it is good, but it is the return (of the results of test).*

(Extract from interview with Nurse 1, Service 1)

Time spent distributing HIVST kits was emphasised, particularly in the ANC clinic. Despite midwives’ positive perceptions of HIVST, they viewed the HIVST offer as an additional task that negatively affected ANC visits.

*We say to ourselves that it’s another addition to our chores, and then it becomes even more (...) it takes time because already, let’s imagine; the explanation itself can already take you 15 minutes; you have to explain it well, there are still the... it can take an hour of time and other women are waiting outside, and grumbling: “This one is not doing her job well. Since I came in this morning...”. She doesn’t know what’s going on inside, and it’s confidential stuff; you’re not going to get the other one in at the same time. You’re obliged to wait. It takes a lot of time frankly; the time is too much.*

(Extract from interview with Midwife 3, Service 1)

To address this issue, midwives advocated for task-shifting to alleviate their workload.

*I don’t know if we could have a special office to do self-testing if, for example, we who are in ANC screen for… we have a case of an STI, if we could have an annex office there so that when we finish with the lady, you go with the lady to the annex office, and then there we do the explanation and everything so that we can receive another lady. Because as long as she is there, we can’t continue our consultation, and in this case even, I think that the case of STI will not escape us because as soon as you have, you know, that there is someone who will take care of it because the person is there for that, especially, it’s his job. So, you, anyway, in our interactions, we will see STI cases; we make our prescription, the prescription for the spouse, and then we call the lady who is there for the self-test, so she takes the lady, and then we take the next one and then the work continues. The work continues, it’s as if there’s no stop; the work continues.*

(Extract from interview with Midwife 2, Service 1)

In the dedicated STI clinic (Service 3), health professionals did not consider HIVST to be an issue. Like his colleagues, one doctor considered HIVST to be a tool that facilitates HIV testing for partners who cannot (or refuse to) come to the health facility.

*On the contrary, it makes our work easier, because before, we were stuck with testing. We had no other alternative; the oral test (HIVST) seems to be indicated for the STI client’s contacts since we can’t reach them. We can give them an oral test via their partners, but we have realised that the oral test can help us even for the patients in front of us, (laughs). So, on the contrary, it makes our job easier (…) When we have a patient in front of us who resists, who doesn’t want to be tested on the spot, you see, so for us, it’s a shot that lightens our work because doing a posttest (counselling); it takes time for the patient to accept. But when you meet these expectations, I’m in a hurry, give me some time, the oral test is offered to him, and it meets these expectations, you see.*

(Excerpt from interview with Doctor 1, Service 3)

Many health professionals mentioned the lack of financial motivation as a limitation to the integration of HIVST into STI consultations. Indeed, the introduction of HIVST was viewed as an additional burden, particularly in Service 1. In addition, the offer of HIVST kits was part of a project, causing several health professionals to expect financial incentives for this additional work. Some health workers believed that the absence of incentives could lead to a lack of interest and involvement in the task of offering HIVST kits.

*There are some midwives who say that they are not paid. (...) There are some who are trained on the job who think that you are motivated, and therefore, they are not motivated to work.*

(Extract from interview with Midwife 2, Service 1)

A few health workers also raised concerns pertaining to the capacity of patients to use the HIVST kits appropriately. They expressed fears regarding the ability of HIVST users to follow the instructions correctly as well as their lack of assistance and accompaniment when performing the test.

*Perhaps for patients who cannot read and write, perhaps, we can fear that they will make a mistake in the use (of the test); if they cannot read or write, well it will be a bit complicated since there is no assistance. That could be it, the illiteracy of a patient. Other than that, I don’t see (any problems).*

(Extract from interview with Doctor 1, Service 3)

One doctor was concerned about the absence of counselling and assistance when performing HIVST.

*For me, the limit of self-testing is that someone gets tested, he has his result in front of him. It’s true, it’s like pregnancy tests, but well, a pregnancy test can be a happy event for the person or not. So, to be unhappy, but in the case of HIV, it’s unhappy that someone gets tested and finds out that they are HIV positive. I don’t know; so, what bothers me about self-testing is the fact that the person is not accompanied when he gets his result.*

(Extract from interview with Doctor 2, Service 2)

### The positive perceptions of the patients who received HIVST for their partners

HIVST was unanimously appreciated by the patients, especially by women, who believed that HIVST was a way to test their partners who refused to go (or were unable to go) to the health facilities for testing. This factor was one of the main reasons why most women accepted the offer of HIVST kits for their partner(s).

*Interviewer: But when you arrived and were offered [the kit] for your partner, what was your reaction?*

*Woman:(Respondent laughs) I say, well, that’s good, well, he’s going to do his test since he himself doesn’t come to the hospital it’s going to help him to do it at home.*

(Interview with Patient 12, Service 3)

HIVST was also appreciated for its reliability, discretion, and ease of use as well as the absence of needles or contact with blood.

*So, when the result came out, I saw that the results are safe; it’s that the test is good because it’s than when you take blood because there are many men who don’t like to give blood. So, I see it as better than giving blood. (...) it’s a test that if you can publish (popularise) it, many men will like it; at least those who want to do it will do it. It’s a very good test.*

*(...) I don’t see any drawbacks. I think it’s great; it’s good.*

(Interview extract with Patient 4, Service 1)

## Discussion

In this qualitative study, we explored the organisation of HIV testing services, as well as the barriers and facilitators associated with the provision of HIVST to STI patients and their partner(s), at three services in Abidjan (Côte d’Ivoire). We found important differences in the organisation of the HIV testing patient flows across services that affected HIV testing and HIVST offers. Comparing the three services showed that, when the HIV testing offer was implemented systematically and task-shifting and good coordination between health professionals where implemented, HIV testing and HIVST offers to patients and their partner(s) were generally more effective. As a whole, HIVST was well-perceived by health professionals and was acceptable to patients.

### For effective and optimal HIVST distribution, we found that well-designed patient flows accompanied by routine offers of HIV testing and task-shifting work best

 In the ANC clinic (Service 1), the offer of HIV testing to women was routine and systematic. The offer of HIVST to the partner(s) of women who were diagnosed with an STI during the ANC consultation was frequent. Midwives were responsible for providing HIV testing and offering HIVST offers for patients’ partner(s). Despite some limitations expressed by the midwives in terms of additional workload, they were, overall, able to offer HIVST.

 In the general health centre (Service 2), despite the existence of task-shifting with respect to HIV testing, offers for HIV testing to patients were not routine, and HIV testing or HIVST offers for partners were not systematic. In addition, there was a lack of coordination between the health personnel who were responsible for STI patient consultations and those who were responsible for HIV testing. Indeed, with the introduction of HIVST, a new patient flow for HIV testing of STI patients was introduced to a preexisting patient flow that was already ill-defined. In this new patient flow for the HIV testing of STI patients, the doctor or nurse who diagnosed the patient with STI was encouraged to present the patient with HIV testing and propose a rapid HIV test or an HIVST. If the patient agreed, he or she was then given a prescription and referred to the care assistant responsible for HIV testing. However, this process did not always happen that way. This inconsistency made it more challenging to provide HIVST kits to patients for their partners’ use.

 In the dedicated STI clinic (Service 3), HIV testing was one of the main activities performed. The patient flow was well defined, including task-shifting for HIV testing. Information on HIV testing and HIVST was provided in the waiting area. There was good coordination between the doctors who consulted with STI patients and the nurses who were responsible for HIV testing. HIV testing and HIVST were often offered to patients and their partners.

Our results showed that the introduction of HIVST into STI consultations could be suboptimal without a good organisation of services. In Services 1 (the ANC clinic) and 3 (which was dedicated to STI consultations), which featured good coordination and a well-defined patients flow, HIV testing, including HIVST offers, was effective. In addition, the health professionals working at these two services received more training or information about HIVST than those in service 2 (the general health centre). This situation influenced the offer of HIVST in these services. Issues related to the absence or inadequacy of training, which affected the quality of services, have previously been addressed in studies in the social sciences [17, 18].

Our observations suggest that when HIV testing was routinely implemented for all patients (in Services 1–2), health professionals were more likely to propose HIV testing to all patients. A systematic review comparing HIV testing approaches found that, in other contexts, opt-out “*testing had higher uptake than opt-in and risk-based testing”* [19]. This approach would not be recommended for low-burden settings in which the focus is on key populations. However, in such a context, it remains relevant to target ANC clinics and health centres dedicated to STI consultations.

In addition to the difficulties related to patient flow and the lack of training of some health professionals (Service 2), the absence of financial incentives for health professionals and the time they were required to spend distributing HIVST kits were emphasised. While all health professionals raised the issue of financial incentives, the time issue was particularly salient in the ANC clinic. In this service, no task-shifting for HIV testing had been implemented. The difficulties highlighted in this context concerning the introduction and offer of HIVST kits reflected the existing dysfunctions in the organisation of health systems [20]. These difficulties include the time requirements of offering HIVST kits on the part of overworked health professionals, which have been reported in other contexts [8], and issues pertaining to the distribution of HIVST kits in particular [21, 22].

Our results confirmed that task-shifting is a good strategy to promote the optimal organisation of HIV testing, in line with WHO recommendations [23]. This is also included to the national health policy in Côte d’Ivoire, specially in the field of reproductive health and HIV treatment [24]. The problem of overloading health professionals, particularly in the context of scarce human resources in sub-Saharan Africa, calls for task-shifting as well as the involvement of community health workers in the management of patients [25, 26]. In the context of HIV testing, HIVST could represent an opportunity to reorganise the services dedicated to testing.

Because of the advantages of HIVST, including time savings and ease of use, it represents an interesting alternative to conventional testing for patients’ partners. Information regarding the use of HIVST can be provided to patients collectively in the waiting room through an awareness video. This approach can even simplify brief pretest counselling, as recently recommended by the WHO [27]. When the provision of information about HIVST is organised in this way, HIVST kits could be offered in the waiting room: this approach would reduce the time required for the provision of such kits as well as the burden on the person responsible for the consultation (the doctor/nurse).

Beyond offering HIVST to patients with STIs, the introduction of HIVST during ANC visits with the goal of reaching STI patients’ partners could be considered if the tasks associated with dispensing HIVST kits are delegated to case assistants, social assistants or people who are exclusively responsible for HIV testing. Studies conducted in other contexts have shown that the introduction of HIVST during ANC visits is feasible and acceptable and that it enables health care providers to reach women’s male partners [28]. To ensure an optimal organisation of the introduction of HIVST into consultations with STI patients, our results suggest: (i) reviewing the overall organisation of testing in health facilities by prioritising services in which HIV testing is routinely offered to patients; (ii) ensuring a good organisation of the patient flow; (iii) involving and training health professionals who are in contact with STI patients; and (iv) ensuring the transfer of tasks related to HIV testing to relieve the burden on doctors, who are generally overworked. From a research perspective, it would be necessary to conduct specific studies to investigate the issue of financial incentives for healthcare workers, which goes beyond the framework of the introduction of HIVST.

### HIVST: an opportunity to test patients’ partner(s) that requires dedicated support

Similar to many other studies, we showed that HIVST was perceived well by health professionals despite some concerns [21, 22]. These fears included the patient’s ability to use HIVST kits properly, the risk of conflict within couples, and the risk of injury. In contrast, a global qualitative systematic review and one of the qualitative ATLAS studies on experiences using and organising HIV self-testing have reported insignificant risks of violence [22, 29].

In addition, because HIVST, through secondary distribution, allows the partner(s) of patients to be reached, it helps health workers who previously encountered difficulties convincing partners to come to health facilities. Indeed, “*adding HIV self-testing to partner notification services can expand the coverage of male partner HIV testing and help to identify those in immediate need of HIV prevention or treatment*” [30].

The patients generally accepted the secondary distribution of HIVST to their partner(s). Most patients, especially women, were motivated by the desire to know the HIV status of their main partner. The acceptability of HIVST among beneficiaries has been shown in several studies [28, 31–35]. Studies conducted in the context of provider-initiated counselling and testing in Côte d’Ivoire have show that patients accept HIV testing when offered. The challenge lies instead in the offer of HIV testing by health professionals, who are either reluctant or do not have sufficient time to do so [7–9].

However, when offering HIVST kits to partners, patients may encounter difficulties, which are related mainly to how HIV testing is discussed with partners; some partners refuse to be tested. These difficulties, far from being specific to HIVST, should be analysed within a more global framework related to the issue of HIV, which remains taboo in the context of the intense stigmatisation of PLHIV [36–39]. In Côte d’Ivoire, as in other West African countries, relations between men and women are still influenced by a patriarchal system that gives little power to women. This result can be analysed in a more global context of gendered and imbalanced power dynamics that impede women’s autonomy, particularly in West Africa [38, 40]. Future studies could also focus on empowering users, especially women, to offer HIVST to their partner(s), by providing better messaging and support options.

### Limitations of the study

It was not possible to observe all patient consultations in the three services as planned in the initial methodology. The two anthropologists were allowed to access the consultation room only when the clinical examination of the patients had been completed, and the health professionals reported an STI diagnosis. Therefore, only the part of the consultation that pertained to the offer of HIV testing was observed: this situation limited the possibility of documenting cases in which HIV testing or HIVST was not offered to patients and their partners. In addition, the presence of the investigator during patient consultations could influence offer of HIV testing, including HIVST, to the patients and/or their partners.

We were able to conduct interviews with only 20/58 patients whose consultations were observed and received an HIVST kit for their partner’s use because most patients who had not yet offered HIVST kits to their partners were either unavailable or unreachable. Nevertheless, we felt that the interviews conducted were sufficiently rich to support an anthropological analysis.

Only two interviews were conducted with partners of STI patients from Service 1, and no interview was conducted with partners of patients from Services 2 and 3. Most patients whose consultations were observed had not yet offered HIVST to their partners. In the context of the study no additional action has been taken to find patients who did not distribute HIVST kits to their partners. In addition, for most patients who agreed to be interviewed, their partner was unavailable, the HIVST kit had not been offered or had not been administered at the time of the interview, or their partner had refused to take the test. For this reason, we removed this category because the data from these 2 interviews were insufficient for further qualitative analyses. As a qualitative work, this study is based on a small number of results. The limitations observed in this study are similar to those observed in other similar studies.

## Conclusion

The results of this study regarding the introduction of HIVST during STI patient consultations revealed disparities in the organisation of services, including the flow of STI patients that can influence the success of the provision of HIVST kits. When HIV testing was “routinised”, the patient flow was well defined, and task-shifting was implemented, the distribution of HIVST kits was more effective and efficient. Structural constraints such as suboptimal organisation of services, nonexistent task-shifting, or health professionals’ lack of financial motivation influenced HIV testing propositions negatively. The introduction of HIVST on its own was not sufficient to overcome these constraints.

Integrating awareness and HIVST demonstration sessions before consultations improves the supply of HIVSTI by reducing the time spent by health professionals during distribution.

Overall, HIVST acceptability was good among health professionals and STI patients who accepted HIVST for their partners’ use. However, the HIVST proposition required time and support to enable patients to propose the use of the kits to their partners on their own. Despite some difficulties that patients were observed to experience when offering the kits to their partners, we observed that the distribution to partners was effective.

The introduction of HIVST through the STI channel is innovative and constitutes a real opportunity to improve access to HIV testing for STI patients and their partners. To achieve successful HIVST integration, we recommend that: (1) health facilities should reorganise their patient flow to be more fluid; (2) they should implement task-shifting where possible; (3) programmes should prioritise HIVST offer services in which HIV testing is routinely offered to patients.

### Supplementary Information

**Additional file 1.** 

**Additional file 2.** 

**Additional file 3.** 

**Additional file 4.** 

**Additional file 5.** 

**Additional file 6.** 

**Additional file 7.** 

**Additional file 8.** 

**Additional file 9.** 

**Additional file 10.** 

**Additional file 11.** 

**Additional file 12.**

## Data Availability

The datasets for this manuscript are not publicly available because of conditions agreed with the participants, but are available from the corresponding author on reasonable request (https://doi.org/10.5281/zenodo.7476751.)

## Abbreviations

ANC: Antenatal care
ATLAS: AutoTest-VIH, Libre d’Accéder à la connaissance de son Statut
HIVST: HIV self-testing
PEPFAR: President’s Emergency Plan for AIDS Relief
PLHIV: People living with HIV
STI: Sexually transmitted infection
SOLTHIS: Solidarité Thérapeutique et Initiatives pour la Santé
UNAIDS: United Nations Programme on HIV/AIDS
WHO: World Health Organisation
