# Supplementary figures and images for: Organisation of testing services, structural barriers and facilitators of routine HIV self-testing during sexually transmitted infection consultations: a qualitative study of patients and providers in Abidjan, Côte d’Ivoire
Source: BMC Infect Dis. 2024 Feb 27;22(Suppl 1):975. doi: 10.1186/s12879-023-08625-x (PMC10900544; doi:10.1186/s12879-023-08625-x)

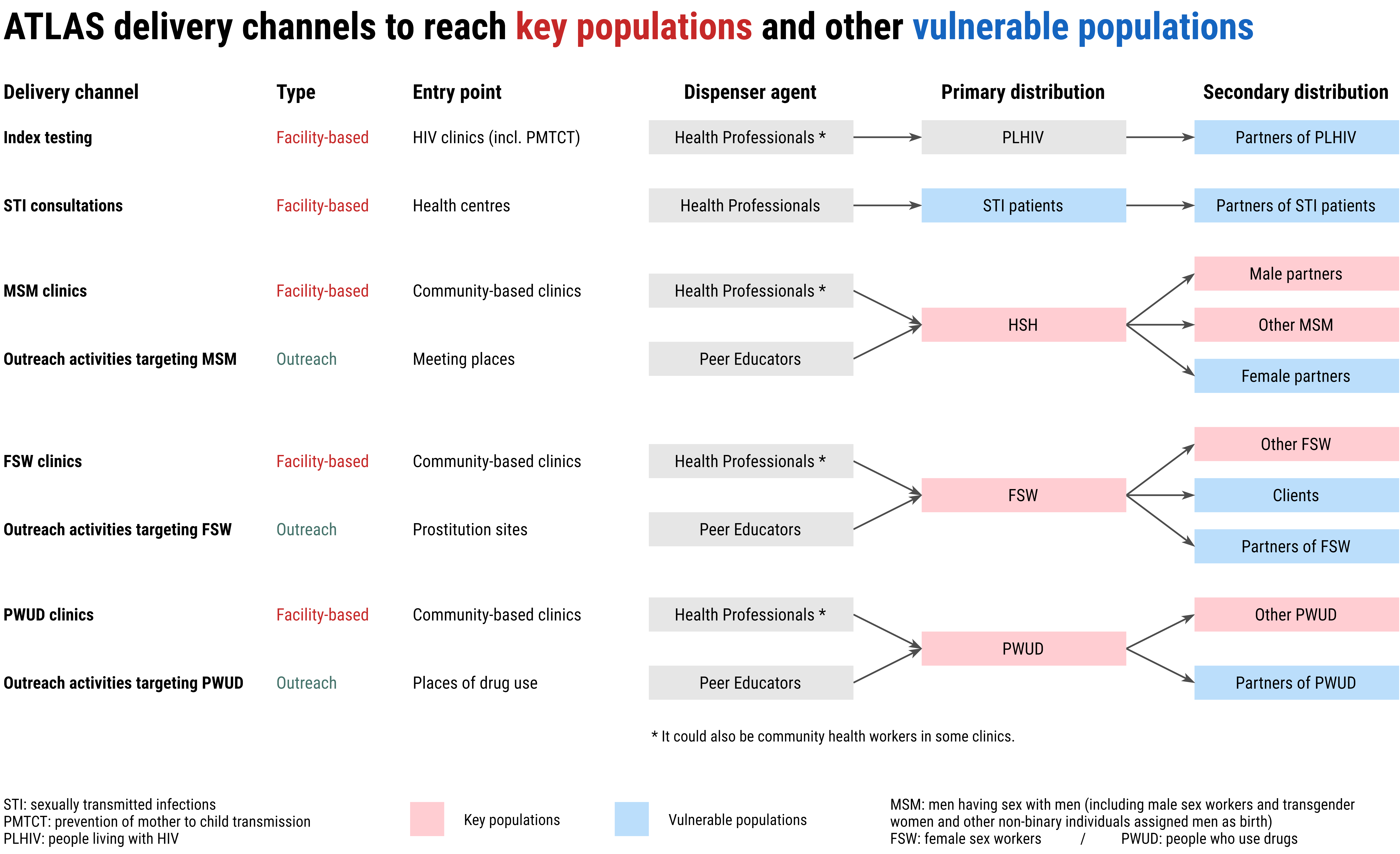

Supplement: Supplementary file 1 — Additional file 1. [file 12879_2023_8625_MOESM1_ESM.png]

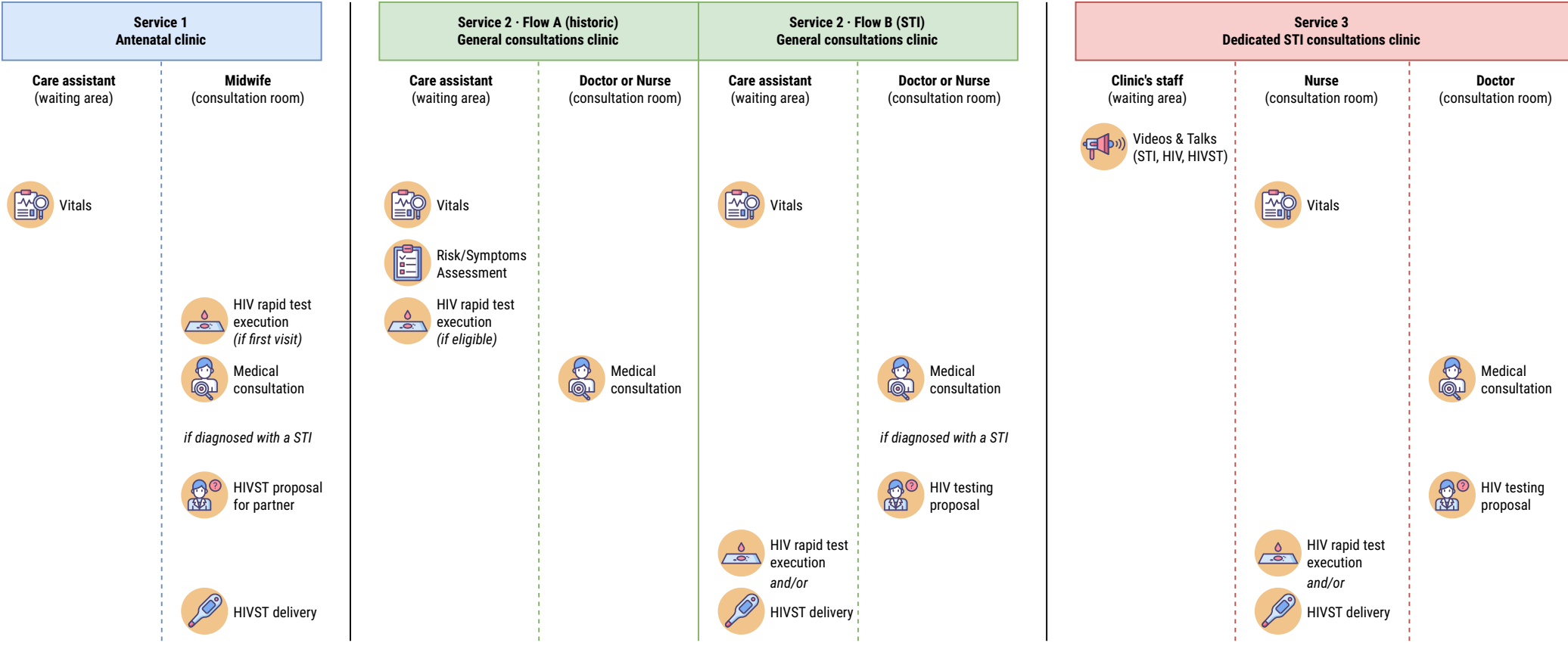

Supplement: Supplementary file 12 — Additional file 12. [file 12879_2023_8625_MOESM12_ESM.pdf]
