# Supplementary material for: Organisation of testing services, structural barriers and facilitators of routine HIV self-testing during sexually transmitted infection consultations: a qualitative study of patients and providers in Abidjan, Côte d’Ivoire
Source: BMC Infect Dis. 2024 Feb 27;22(Suppl 1):975. doi: 10.1186/s12879-023-08625-x (PMC10900544; doi:10.1186/s12879-023-08625-x)
Supplement: Supplementary file 6 — Additional file 6. [file 12879_2023_8625_MOESM6_ESM.pdf]

6-Table 3: Information of health professionals

Pseudo	Service	Age-range	Sex	Education level	Profession	Self-test distribution training by Solthis
Midwife 1	Service 1	36-40	Woman	High	Midwife	Full training
Midwife 2	Service 1	41-45	Woman	High	Midwife	Full training
Midwife 3	Service 1	31-35	Woman	High	Midwife	Full training
Nurse 1	Service 2	26-30	Man	High	Nurse	Full training
Nurse 2	Service 2	46-50	Woman	High	Nurse	No training
Nurse 3	Service 2	36-40	Man	High	Nurse	No training
Nurse 4	Service 2	36-40	Man	High	Nurse	No training
Clinical advisor	Service 2	36-40	Woman	Secondary	Clinical advisor	Full training
Social worker	Service 2	31-35	Woman	High	Social worker	Full training
Care assistant	Service 2	31-35	Woman	Secondary	Care assistant	Training on site
Doctor 1	Service 2	46-50	Man	High	Doctor	Full training
Doctor 2	Service 2	41-45	Woman	High	Doctor	No training
Doctor 3	Service 2	41-45	Woman	High	Doctor	No training
Nurse 1	Service 3	41-45	Woman	High	Nurse	Full training
Nurse 2	Service 3	41-45	Woman	High	Nurse	Make-up training
Doctor 1	Service 3	46-50	Man	High	Doctor	Make-up training
Doctor 2	Service 3	41-45	Man	High	Doctor	Make-up training
Doctor 3	Service 3	36-40	Woman	High	Doctor	Make-up training
