# Supplementary material for: Organisation of testing services, structural barriers and facilitators of routine HIV self-testing during sexually transmitted infection consultations: a qualitative study of patients and providers in Abidjan, Côte d’Ivoire
Source: BMC Infect Dis. 2024 Feb 27;22(Suppl 1):975. doi: 10.1186/s12879-023-08625-x (PMC10900544; doi:10.1186/s12879-023-08625-x)
Supplement: Supplementary file 7 — Additional file 7. [file 12879_2023_8625_MOESM7_ESM.pdf]

7-Observation guides for the study of the HIV testing proposal for STI patients

Observation Guide-Consultation

General information

Location

Time

Duration

Persons present

HIVST proposal for the partner

Circumstances: “routine” or specific consultation

Information provided, terms used

Patient reactions

Questions asked

Acceptance, refusal, request for time to think, more information?

Reasons expressed

When the HIVST kit is delivered

Description of the kit

Description of the information provided

Description of the questions asked/reactions of the patient

Social report

Terms and conditions of exchanges

Attitudes and gestures

Observation Guide - Meeting sites

General information

Location

Type of activity

Time

Duration

Persons present

Specific information about the activity

Objective of the activity/order of the day

Profile of the participants

Questions about HIV and AIDS

Are issues regarding HIV and AIDS addressed during staff meetings?

By whom?

How are they approached?

Participants' reactions

Positive/negative points discussed

Difficulties in the clinic?

Negotiations on the terms and conditions of the dispensing of HIVST kits, the people to whom the self-test should be offered, the information to be provided, etc.

Discussions on HIV partner testing and how to provide self-tests to partners

Discussions on confidentiality issues
