# Supplementary material for: Organisation of testing services, structural barriers and facilitators of routine HIV self-testing during sexually transmitted infection consultations: a qualitative study of patients and providers in Abidjan, Côte d’Ivoire
Source: BMC Infect Dis. 2024 Feb 27;22(Suppl 1):975. doi: 10.1186/s12879-023-08625-x (PMC10900544; doi:10.1186/s12879-023-08625-x)
Supplement: Supplementary file 8 — Additional file 8. [file 12879_2023_8625_MOESM8_ESM.pdf]

8-Table 1: Information of patients whose consultations were observed

Service	Sex	Consultation reason	Marital status	Age range	Total
Maternity/ANC	Woman	ANC[1]	In a Relationship	25-49	29
Maternity/ANC	Woman	ANC	NI[2]	25-49	
Maternity/ANC	Woman	ANC	Married	<24	
Maternity/ANC	Woman	ANC	Married	25-49	
Maternity/ANC	Woman	ANC	Married	25-49	
Maternity/ANC	Woman	ANC	Married	25-49	
Maternity/ANC	Woman	ANC	NI	25-49	
Maternity/ANC	Woman	ANC	NI	<24	
Maternity/ANC	Woman	ANC	Married	25-49	
Maternity/ANC	Woman	ANC	Married	25-49	
Maternity/ANC	Woman	ANC	NI	25-49	
Maternity/ANC	Woman	ANC	In a Relationship	<24	
Maternity/ANC	Woman	ANC	NI	<24	
Maternity/ANC	Woman	ANC	Married	25-49	
Maternity/ANC	Woman	ANC	Married	25-49	
Maternity/ANC	Woman	ANC	NI	25-49	
Maternity/ANC	Woman	ANC	Married	<24	
Maternity/ANC	Woman	ANC	Married	25-49	
Maternity/ANC	Woman	ANC	Married	25-49	
Maternity/ANC	Woman	ANC	Married	<24	
Maternity/ANC	Woman	ANC	Married	25-49	
Maternity/ANC	Woman	ANC	In a Relationship	<24	
Maternity/ANC	Woman	ANC	In a Relationship	<24	
Maternity/ANC	Woman	ANC	In a Relationship	25-49	
Maternity/ANC	Woman	ANC	In a Relationship	25-49	
Maternity/ANC	Woman	ANC	In a Relationship	<24	
Maternity/ANC	Woman	ANC	In a Relationship	<24	
Maternity/ANC	Woman	ANC	In a Relationship	<24	
Maternity/ANC	Woman	ANC	NI	25-49	
General Clinic	Woman	RFS[3]	Single	25-49	16
General Clinic	Woman	Reference for HIV rapid test	Single	25-49	
General Clinic	Woman	STI	Single	<24	
General Clinic	Woman	Reference for HIV rapid test	Single	<24	

[1] Antenatal clinic visits

[2] No Information

[3] Reference for screening

General Clinic	Woman	STI	In a Relationship	25-49	
General Clinic	Man	Reference for HIV rapid test	Single	25-49	
General Clinic	Man	STI	Single	<24	
General Clinic	Woman	STI	NI	<24	
General Clinic	Woman	STI	NI	<24	
General Clinic	Man	STI	Single	25-49	
General Clinic	Woman	STI	Single	25-49	
General Clinic	Man	Other	Married	25-49	
Clinic	Man	STI	Married	25-49	
General Clinic	Woman	STI	NI	25-49	
General Clinic	Woman	STI	In a Relationship	<24	
General Clinic	Woman	STI	Married	25-49	
STI Clinic	Woman	STI	In a Relationship	25-49	
STI Clinic	Man	STI	In a Relationship	25-49	
STI Clinic	Woman	STI	In a Relationship	<24	
STI Clinic	Man	STI	In a Relationship	25-49	
STI Clinic	Woman	STI	In a Relationship	25-49	
STI Clinic	Woman	STI	In a Relationship	25-49	
STI Clinic	Man	STI	Married	25-49	
STI Clinic	Woman	STI	In a Relationship	25-49	
STI Clinic	Woman	STI	Single	25-49	
STI Clinic	Woman	STI	In a Relationship	25-49	
STI Clinic	Man	STI	Married	25-49	
STI Clinic	Woman	STI	Single	<24	
STI Clinic	Man	STI	In a Relationship	25-49	
STI Clinic	Man	STI	In a Relationship	25-49	
STI Clinic	Man	STI	Married	25-49	
STI Clinic	Woman	STI	Single	<24	53
STI Clinic	Woman	STI	Married	25-49	
STI Clinic	Woman	STI	In a Relationship	<24	
STI Clinic	Woman	STI	In a Relationship	25-49	
STI Clinic	Woman	STI	In a Relationship	25-49	
STI Clinic	Woman	STI	In a Relationship	25-49	
STI Clinic	Woman	STI	In a Relationship	25-49	
STI Clinic	Man	STI	In a Relationship	25-49	
STI Clinic	Man	STI	In a Relationship	25-49	
STI Clinic	Man	STI	Married	25-49	
STI Clinic	Man	STI	In a Relationship	25-49	
STI Clinic	Man	STI	Married	25-49	
STI Clinic	Woman	STI	Married	25-49	
STI Clinic	Man	STI	Single	<24	
STI Clinic	Woman	STI	In a Relationship	25-49	

STI Clinic	Man	STI	In a Relationship	25-49	
STI Clinic	Woman	STI	Married	25-49	
STI Clinic	Man	STI	In a Relationship	25-49	
STI Clinic	Man	STI	Single	25-49	
STI Clinic	Woman	STI	In a Relationship	<24	
STI Clinic	Woman	STI	In a Relationship	25-49	
STI Clinic	Man	STI	Married	25-49	
STI Clinic	Man	STI	In a Relationship	25-49	
STI Clinic	Woman	STI	In a Relationship	25-49	
STI Clinic	Man	STI	In a Relationship	25-49	
STI Clinic	Man	STI	In a Relationship	25-49	
STI Clinic	Woman	STI	In a Relationship	25-49	
STI Clinic	Woman	STI	In a Relationship	25-49	
STI Clinic	Woman	STI	In a Relationship	25-49	
STI Clinic	Man	STI	Single	25-49	
STI Clinic	Woman	STI	In a Relationship	<24	
STI Clinic	Man	STI	Single	25-49	
STI Clinic	Man	STI	In a Relationship	25-49	
STI Clinic	Woman	STI	In a Relationship	25-49	
STI Clinic	Man	STI	In a Relationship	25-49	
STI Clinic	Woman	STI	Married	25-49	
STI Clinic	Man	STI	Married	25-49	
STI Clinic	Woman	STI	In a Relationship	25-49	
