# Supplementary material for: Organisation of testing services, structural barriers and facilitators of routine HIV self-testing during sexually transmitted infection consultations: a qualitative study of patients and providers in Abidjan, Côte d’Ivoire
Source: BMC Infect Dis. 2024 Feb 27;22(Suppl 1):975. doi: 10.1186/s12879-023-08625-x (PMC10900544; doi:10.1186/s12879-023-08625-x)
Supplement: Supplementary file 9 — Additional file 9. [file 12879_2023_8625_MOESM9_ESM.pdf]

9-Table 2: Information of patients who participated in the interviews

Pseudo	Sex	Marital status	Profession	Age range	Education level	HIVST receipt for ego	HIVST receipt for partner	HIVST proposed to partner	Partner's reaction	HIVST use
P[1]2 Service 1	Woman	In a Relationship	Informal work	16-20	Secondary	No	Yes/1	Yes	Accepted	Completed
P3Service1	Woman	In a Relationship	Informal work	31-35	Primary	No	Yes/1	Yes	Accepted	Completed
P4Service1	Woman	In a Relationship	Elève	16-20	Secondary	No	Yes/1	Yes	Refused	Completed
P5Service1	Woman	In a Relationship	Informal work	21-25	Primary	No	Yes/1	Yes	Accepted	No
P6Service1	Woman	Mared	Informal work	31-35	Primary	No	Yes/1	Yes	Accepted	Completed
P7.Service2	Woman	In a Relationship	Public servant	31-35	Secondary	No	Yes/1	No	NA	NA
P1Service2	Woman	In a Relationship	NI	16-20	Secondary	No	Yes/1	No	NA	No
P1Service 3	Woman	In a Relationship	Student	21-25	Higher	No	Yes/1	Yes	Accepted	Completed
P2Service 3	Man	In a Relationship	Public servant	36-40	Higher	No	Yes/1	Yes	Accepted	No
P3Service 3	Man	In a Relationship	Public servant	36-40	Higher	No	Yes/3	Yes	Accepted	2/3 Completed
P4Service 3	Woman	In a Relationship	Student	31-35	Higher	No	Yes/1	Yes	Refused	No
P5Service 3	Woman	In a Relationship	Informal work	36-40	Higher	No	Yes/1	Yes	Accepted	No
P6Service 3	Woman	In a Relationship	Informal work	21-25	Secondary	No	Yes/1	Yes	Accepted	Completed
P7Service 3	Woman	In a Relationship	Student	26-30	Higher	No	Yes/1	Yes	Refused	No
P8Service 3	Woman	In a Relationship	Public servant	26-30	Higher	No	Yes/1	Yes	Accepted	Completed
P9Service 3	Man	In a Relationship	Public servant	46-50	Higher	Yes/1	No	NA	NA	NA
P10Service 3	Man	mared	Public servant	46-50	Secondary	No	Yes/1	Yes	Accepted	Completed
P11Service 3	Woman	mared	Public servant	36-40	Higher	No	Yes/1	Yes	Accepted	No
P12Service 3	Woman	In a Relationship	Public servant	36-40	Higher	No	Yes/1	Yes	Refused	No
P13Service 3	Woman	In a Relationship	Informal work	41-45	Higher	No	Yes/1	Yes	Refused	No

[1] P=Patient
