# Supplementary material for: Organisation of testing services, structural barriers and facilitators of routine HIV self-testing during sexually transmitted infection consultations: a qualitative study of patients and providers in Abidjan, Côte d’Ivoire
Source: BMC Infect Dis. 2024 Feb 27;22(Suppl 1):975. doi: 10.1186/s12879-023-08625-x (PMC10900544; doi:10.1186/s12879-023-08625-x)
Supplement: Supplementary file 10 — Additional file 10. [file 12879_2023_8625_MOESM10_ESM.pdf]

10-Thematic interview guide - health personnel

THEMES	**QUESTIONS**
General information	• Age<br>• Professional training<br>• Place of work<br>• Professional activity
Activities and management of HIV and STI patients	• Description of department activities (tasks and patient flow)<br>• Involvement in the management of HIV<br>• Management and follow-up of STI patients
HIV testing offer for STI patients' before HIVST	• Before HIVST introduction, did you offer testing to patients who presented with STIs?<br>• If so, what HIV testing strategies were in place at your facility to screen STI patients and their partners?<br>• What difficulties do you face in HIV testing proposal for STI patients and their partners?
Setting up HIVST	• When and how was HIVST set up for STI patients and their partners?<br>• What was the mechanism mobilised to set up HIVST?<br>• What is your level of involvement in setting up the HIVST?<br>• Have you received training on HIVST? If yes, when? How many times? By whom?<br>• What did you learn about the dispensing of HIVST kits, particularly the dispensing channels?<br>• Have you noted any difficulties, limitations or strengths in the implementation of HIVST?
HIVST and dispensing HIVST kits to patients for their partners	• On what basis do you select the STI patients to whom you dispense HIVST kits?<br>• Can you describe how you dispense HIVST kits to STI patients themselves and to their partners (content of information presented to them, terms used to present HIVST kits, time...)?
Attitudes of STI patients towards the offer of HIVST for themselves and their partners	• Can you describe the reactions of STI patients to the offer of HIV treatment (refusal/acceptance/hesitation/questions)?<br>• What are the reactions of STI patients and partners to the results after testing? (Do they inform the clinic about the results? How? [By phone, face to face, accompanied, indirect information via the partner] What is the timeframe?)
Attitudes of STI patients and/or partners after HIVST	• Do patients confirm their status if they receive a reactive result following HIVST?<br>• What is the role of the STI patient in accompanying his or her partner?<br>• What is the caregiver-patient relationship when HIVST is performed?<br>•
Perceptions and appreciation of HIVST kits and their dispensing	• How do you feel about HIVST? (List strengths, limitations...)

among STI patients and their partners	• How do you like the delivery of HIVST through this channel? (List benefits, limitations, fears, difficulties, workload...)
Suggestions and recommendations for the dispensing of HIVST kits to STI patients and their partners	• Do you have any recommendations for better distribution and use of HIVST in the general population and in particular to STI patients?<br>• Do you have any questions?<br>• What is your final word?<br>• Acknowledgement
