# Supplementary material for: Organisation of testing services, structural barriers and facilitators of routine HIV self-testing during sexually transmitted infection consultations: a qualitative study of patients and providers in Abidjan, Côte d’Ivoire
Source: BMC Infect Dis. 2024 Feb 27;22(Suppl 1):975. doi: 10.1186/s12879-023-08625-x (PMC10900544; doi:10.1186/s12879-023-08625-x)
Supplement: Supplementary file 11 — Additional file 11. [file 12879_2023_8625_MOESM11_ESM.pdf]

11-Thematic interview guide – STI patients

THEMES	**QUESTIONS**
General information	• Age (ego)<br>• Sex (ego and partner(s))<br>• Profession (ego and partner(s))<br>• Place of residence (ego and partner(s))<br>• Family environment, marital status, number of children<br>• Education/Level of schooling (ego and partner(s))<br>• Religion (ego and partner(s))<br>• Ethnicity (ego and partner(s))
Knowledge of STIs:<br>*Sources of knowledge*<br>*Manifestations*<br>*Modes of transmission*<br>*Prevention/treatment/cure*	• Can you tell me where you first heard about sexually transmitted infections (STIs)? (Probe for knowledge of STIs)<br>• What are some local terms used for STIs?<br>• Can you give me names of STIs?<br>• What are the manifestations of the disease (STIs)?<br>• What are the modes of transmission?<br>• How can I prevent or protect myself?<br>• Are there any treatments for STIs?<br>• Can an STI be cured?
STI prevention and consultation	• What preventive measures do you take to avoid the risk of contracting STI?<br>• Have you ever had an STI consultation (before last visit)? If yes, when? How many times?<br>• Reasons and circumstances of the consultation?<br>• Were you offered HIV testing as a result of the visit?<br>• Did you tell your partner(s) about the visit?
Care Itinerary	• Can you describe your care itinerary when you felt the symptoms (self-medication, direct consultation at a health facility, healer?)<br>• If you self-medicate, can you give me the names of the drugs or products used?<br>• Did you feel any improvement after using these products?<br>• If you used a formal health facility, can you tell us about your consultation (diagnosis of the health care provider, treatment proposal (with partner?), follow-up...)<br>• If you used a traditional healer, can you tell us about your consultation (diagnosis, proposed treatment, use...)
Knowledge of HIV:<br>*Sources of Knowledge*<br>*Manifestations*	• Can you tell me where you first heard about HIV?<br>• What are the manifestations of the disease?<br>• What are the modes of transmission?<br>• How can you prevent it?

Modes of transmission<br>*Prevention/treatment/cure*	• Is there a treatment for HIV?<br>• Is there a cure for HIV?
HIV prevention and testing practices (pre-HIVST):<br>*Attitudes towards HIV risk*<br>*Testing practices*<br>*Information on the serological status of family members/partners*<br>*Communication about HIV*	• What prevention measures do you take to avoid the risk of HIV transmission?<br>• Have you ever been tested for HIV? If so, how many times? Why and under what circumstances have you been tested?<br>• Do you know the HIV status of your family/partner(s)?<br>• Do you ever discussed HIV with your partner(s)?
Perceived risk of HIV infection	• Do you think you are at risk for HIV?<br>• If so, why?<br>• Are there any people who might be at greater risk of HIV infection? If so, who and why?<br>• Do you think people with an STI might be at greater risk of getting HIV?
Knowledge about HIVST:<br>*Sources of knowledge about HIVST-AIDS*<br>*Knowledge Content*	• Have you heard of HIV self-testing (before proposal)?<br>• If so, where and by whom?<br>• What do you know about HIVST?
Provider's HIVST proposal to the patient *(How it was proposed by the provider)*	• Can you describe you were offered to deliver an HIVST your partner, the content of the information, the medium, and the terms used?<br>• What did you understand about HIVST? (Probe the patient's understanding of the way the HIVST was presented to him or her (objective, use, note the terms used...))<br>• How did you react to the proposal?<br>• Can you describe your acceptance/refusal to offer the test to your partner?<br>• What were your reasons, motivations, expected benefits or limitations?
Proposal of the HIVST to his or her partner(s) *(How the patient proposed HIVST to his or her partner)*	If accepted:<br>• To which partner (if multiple partners)?<br>• What is your relationship with the partner(s) (married, cohabitating, occasional partner)<br>• How was the proposal made to the partner (time, place, content of the information, medium, terms used, etc.)?<br>• What were the reactions of the partner: acceptance of the self test, refusal (why?), time needed for reflection, search for additional information, from whom (including internet)?
Practices and usage	• Self-testing practice: Can you describe where and how the HIV test took place and whether it took place with your partner or alone?<br>• Ease/difficulty of use? Ease/difficulty of understanding the test result?<br>• Test result: Did you share the information with your partner? With other people?

	• Perception of the test results: Did you think the test was reliable, did you have doubt?<br>• Steps taken after the test, depending on the result. If reactive result, what did you do (call the hotline?) Medical consultation (alone or accompanied by the partner?), implementation of a follow-up, treatment...
Perceptions of HIVST and the impact of HIVST:<br>*On the relationship: trust, communication, distance, violence, sexuality...*<br>*On access to and relationship with care (especially if the result is positive)*<br>*On HIV testing practices, prevention practices, relationship with HIV, risk...*	• How do you feel about HIVST?<br>• What impact has the use of HIVST had on your relationship with your partner(s)? (Depending on the results)<br>• If positive: what was the attitude of your partner(s) when sharing your HIV status? (Probe for trust, support or rejection, break-up, violence, stigma...)<br>• Have you discussed with your partner(s) treatment, prevention practices to avoid the risk of transmission/or "overcontamination" (if partner is also HIV positive)
Recommendations, suggestions, questions, final comments	• Do you have any recommendations for better distribution and use of HIVST in the general population, especially for those who refuse or do not have access to conventional tests?<br>• Do you have any questions?<br>• Do you have any final comments?<br>• Acknowledgements
